# Design of 2.45 GHz High-Efficiency Rectifying Circuit for Wireless RF Energy Collection System

**DOI:** 10.3390/mi15030340

**Published:** 2024-02-29

**Authors:** Yanhu Huang, Jiajun Liang, Zhao Wu, Qian Chen

**Affiliations:** 1 Guangxi University Key Lab of Complex System Optimization and Big Data Processing, Guangxi Applied Mathematics Center, Yulin Normal University, Yulin 537000, China; yanhuhuang@ylu.edu.cn (Y.H.); kianty@ylu.edu.cn (Z.W.); 19899218213@163.com (Q.C.); 2 School of Electronic and Information Engineering, South China University of Technology, Guangzhou 510641, China

**Keywords:** energy collection, rectifying circuit, receiving antenna, high efficiency

## Abstract

A 2.45 GHz high-efficiency rectifying circuit for a wireless radiofrequency (RF) energy collection system is proposed. The RF energy collection system is composed of a transmitting antenna, a receiving antenna, a rectifying circuit and a load. The designed receiving antenna is a kind of dual-polarised cross-dipole antenna; its bandwidth is 2.3–2.5 GHz and gain is 7.97 dBi. The proposed rectifying circuit adopts the technology of an output matching network, which can suppress the high-harmonic components. When the input power at 2.45 GHz is 13 dBm and the load is 2 kΩ, the highest conversion efficiency of RF-DC is 74.8%, and the corresponding maximum DC output voltage is 4.92 V. The experiment results are in good agreement with the simulation results, which shows a good application prospect.

## 1. Introduction

With the rapid development of wireless communication technology, Wireless-Fidelity (WiFi), Bluetooth, the Internet of Things (IoT) and other communication technologies have entered into most areas of people’s lives, and these mature technologies have brought a lot of convenience to people’s lives. Currently, 5G mobile communication is being rapidly developed and promoted, and the era of the Internet of Things is coming; and the future 6G communication is also in full swing in academic research. This means that in the 5G and 6G era, the electromagnetic environment of people’s living space will become more and more complex, and electromagnetic waves will be more densely distributed in all corners. Electromagnetic waves are both carriers of information transmissions and energy at the same time. How to effectively collect and utilize the electromagnetic waves in the environment are effective considerations for alleviating the problem of energy shortage, and it is also the development trend of the new institutional communication technology for the future. Microwave energy transmission and collection technology has been a research hotspot in recent years; short-distance wireless energy transmission technology has been commercially applied in the fields of new energy vehicles, cell phones, laptops, etc., while medium- and long-distance wireless energy transmission technology has not been popularized due to the existence of a number of unresolved technical problems. Wireless energy transmission technology is mainly composed of four components: a microwave transmitter, microwave transmitter antenna, microwave receiver antenna and microwave rectifier circuit, which involve key technologies including high-efficiency DC-RF conversion technology, a high-power transmitter antenna, a high-efficiency receiver antenna and a high-efficiency receiver rectifier circuit.

With the rapid development of information technology, consumer electronic products are widely used in daily life. In order to maintain the continuous and uninterrupted operation of these devices, a continuous energy supply must be provided. The conventional battery power supply method has the problems of a short service life and the high cost of battery replacement. Based on this, wireless RF energy collection technology is considered to be a better alternative method for the energy supply [1].

A wireless RF energy collection system is the form of collecting RF energy at different frequencies in the natural environment through a receiving antenna first, and then converting into DC energy through a rectifying circuit. As a new energy collection technology, it has great application value in the fields of low-power wireless sensor networks [2], biomedical sensors [3,4], Internet of Things mobile terminal devices [5,6,7], intelligent wearable devices [8,9,10,11,12], and so on. The traditional wireless RF energy collection system mainly includes an RF signal source, transmitting antenna, receiving antenna and rectifying circuit. The basic composition of the rectifying circuit mainly includes an input filter, a rectifying diode, a pass-through filter and a DC load. The ratio of the DC power output of the rectifying circuit to the received RF signal power is called the rectification efficiency or the conversion efficiency, which is the most important index in evaluating the performance of a wireless RF energy collection system.

In the rectifying circuit of the RF energy collection system, due to the nonlinear characteristics of the rectifying diode, high-harmonic components are generated, which will flow back to the antenna for radiation again, resulting in energy loss. In the traditional rectifying circuit, the low-pass filter is used to suppress the high-harmonic components generated by the nonlinear rectifying diode to improve the conversion efficiency of the rectifying circuit. A broadband matching network is used in some wide input power rectifying circuits to increase the conversion efficiency [13,14,15].

According to existing research results, with an input power greater than 15 dBm, a rectification efficiency of more than 50% can be obtained only in a relatively narrow bandwidth range. Once the optimal maximum input power value is exceeded, the rectification efficiency will decrease sharply [16,17]. In the actual electromagnetic environment, the wireless microwave signal is dynamic change. Therefore, when the power of the RF signal is received by the receiving antenna changes, the rectification circuit should also be required to maintain a stable conversion efficiency. In [18], a miniaturized RF energy collector for an Internet of Things (IoT) application was proposed. Considering the insensitivity to the ambient RF energy and the low-profile design, the proposed RF energy collector uses a two-layer printed circuit board (PCB) substrate consisting of an orthogonally deployed antenna with an LC balancer, impedance matching and a Dickson charge pump circuit. The double-polarized antenna adopts the bow-typed dipole antenna. The conversion efficiency of the rectifying circuit is up to 71.4% from any incident ambient RF signal. In [19], in order to solve the problem of a low and non-uniform electromagnetic energy density in the environment, a triband monopole rectifying antenna was designed. A single diode rectifier was selected to operate efficiently over a wide range of input power levels from −10 dBm to 5 dBm and provide a minimum conversion efficiency of 60%. In [20], a broadband circularly polarized rectifying antenna was proposed, and a wideband rectifier with branch coupler was designed, with a bandwidth of 1.7 to 2.6 GHz in a wide operating power and a wide output load range. The RF–DC conversion efficiency at the ambient wireless power level was effectively improved. In this rectifier, the peak efficiency was 69% at 12 dBm (at 1.85GHz) and 64% at 10 dBm (at 2.45 GHz).

There have been many related reports in the field of broadband high-efficiency rectifier circuit technology; however, the difficulty between the operating bandwidth and conversion efficiency has still not been completely solved. In [21], a single-layer coplanar waveguide wideband rectifier circuit was described, which uses a voltage multiplier tube connected in series with a wideband matching network, and in order to realize miniaturization, the wideband matching consists of a series dual-inductor lumped element that finally achieves a conversion efficiency of more than 45% in the frequency range of 0.1–2.5 GHz; although the method obtains a wide operating bandwidth, the in-band conversion efficiency is not high. In [22], a novel three-stage impedance matching technique was used to realize a compact broadband high-efficiency rectifier circuit. The technique first utilizes a linearly tapered transmission line to achieve impedance modulation for different input power levels, and then further uses a second-order circular impedance matching branch to achieve the final broadband impedance matching. Ultimately, the technique achieves a conversion efficiency higher than 50% at an input power of 10 dBm and a frequency range of 0.97–2.55 GHz. A compact broadband high-efficiency energy harvesting rectifier has been proposed in the literature [23]. In this structure, a high conversion efficiency over a wide frequency range was achieved by designing a compact broadband low-loss matching network and a direct current filter with a wide stopband. For the broadband low-loss matching network, low loss and compact dimensions were achieved by strategically selecting and designing three transmission line segments. The final rectifier bandwidth obtained was 41.5% (2.0–3.05 GHz), and the RF–DC power conversion efficiency was higher than 70% at an input power of 10 dBm; when the input power decreases to 0 dBm, the measured efficiency stays above 45% in the range of 1.9–3.05 GHz, and at an input power of 14 dBm, the maximum measured efficiency is 75.8%. In [24], a circularly polarized rectifier antenna that has the advantage of high efficiency over a wide range of input power and frequency was presented. The antenna consists of an efficient rectifier and a broadband circularly polarized antenna. In this rectifier, the matching performance of the circuit over a wide input power range and frequency range was greatly improved by introducing a new broadband impedance compression technique. Simulation and measurement results showed that the conversion efficiency reached more than 60% (up to 76%) in the input power range of 5–17 dBm and the frequency band of 1.7–2.9 GHz (mobile, Wi-Fi, and ISM bands), which is a significant improvement in the performance of this rectifier circuit in the two metrics of bandwidth and efficiency. In summary, although there have been scholars trying to overcome the bottleneck between the operating bandwidth and conversion efficiency of rectifier circuits, the existing techniques and methods have not been able to completely solve the problem.

In order to improve the receiving efficiency and obtain a relatively high conversion efficiency in the wide input power, a wireless RF energy collection system working at 2.45 GHz was studied and designed in this study. The receiving antenna is a kind of a dual-polarized cross-dipole antenna. In order to facilitate the integration with the receiving antenna and obtain a higher conversion efficiency, the proposed rectifying circuit uses the form of diode voltage doubling and lumped elements to build the matching filter network, so as to suppress the harmonic energy and improve the rectification efficiency. Finally, the whole system was processed and tested. The test results show that the rectification circuit has a good harmonic suppression effect. At the input power of 13 dBm, the maximum conversion efficiency of the rectifier antenna reaches 74.8%, and the conversion efficiency is larger than 40% in the input range of 0–18 dBm.

## 2. Rectifying Circuit Simulation and Design

**Figure 1:**
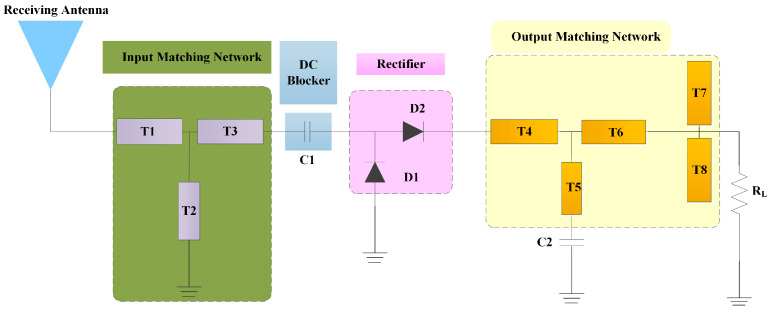
A schematic of the proposed wireless RF energy collection system.

Figure 1 is a schematic of the proposed wireless RF energy collection system; it mainly includes a receiving antenna and rectifying circuit. The basic composition of the rectifying circuit mainly includes an input matching network, rectifying diode (rectifier), output matching network and load. The two series microstrip branches, T1 and T2, and one parallel shorter branch, T3, constitute the H-typed input matching network. As seen, T1 is a 50 Ω microstrip line connected to the receiving antenna, and T2 is connected to the ground. The input impedance can be optimized by changing the sizes of the branches. A block capacitance C_1_ is used to isolate the DC signal between the rectifying diode and the input port. Meanwhile, only the high-frequency signals are allowed to pass through the rectifying diode. Rectifying diode HSMS-286C is used in this circuit. HSMS-286C is composed of two HSMS-2860 diodes inside the same package, which are electrically connected in parallel. This kind of rectifying diode can achieve twice the rectification effect compared to a single diode.

An output matching network is used at the end of the circuit, which consists of an output matching circuit and a bypass capacitance C_2_. The output matching network consists of two series microstrip lines, T4 and T6, a parallel short-circuited microstrip line, T5, which is connected in series with capacitor C_2_, and two parallelly connected open-circuited microstrip lines, T7 and T8, at the end. By reasonably adjusting the sizes of these microstrip lines, a better matching can be obtained at a frequency of 2.45 GHz. The optimal value of C_1_ is 33 pF, C_2_ is 22 pF, and the load *R* is 2000 Ω.

**Figure 2:**
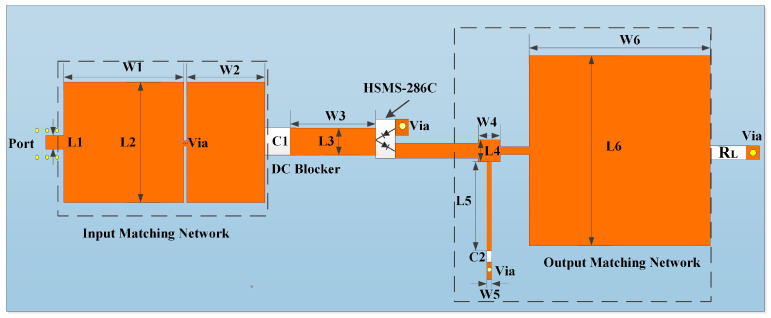
The layout of the proposed rectifying circuit.

Figure 2 shows the layout of the proposed rectifying circuit. Simulation software ADS2021 was used to simulate the circuit model. The printed circuit board selected for the rectifier circuit was Rogers 4350b, with a dielectric constant of 3.48, a loss angle of 0.017 and a thickness of 1.524 mm. The optimized parameters of the proposed rectifying circuit are shown in Table 1.

**Figure 3:**
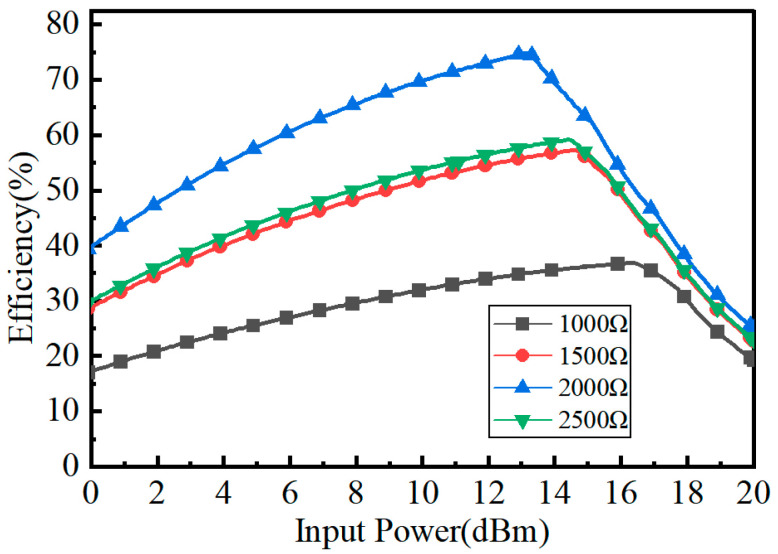
Rectifier efficiency versus input RF power *P_in_*.

As seen in Figure 3, the simulated conversion efficiency is changed significantly with the load. When the input power *P_in_* is 13 dBm and the load *R_L_* is 1000 Ω, the RF-to-DC conversion efficiency is 30%; under the same input power of 13 dBm with a load *R* of 2000 Ω, the RF-to-DC conversion efficiency is 74.8%. Therefore, it can be obtained that the output DC power of the rectifying circuit mainly depends on the output voltage and the output load, and the output load of the rectifying circuit is one of the key parameters affecting the overall conversion efficiency. The load can be adjusted to increase and improve the conversion efficiency within the predefined input power range.

**Figure 4:**
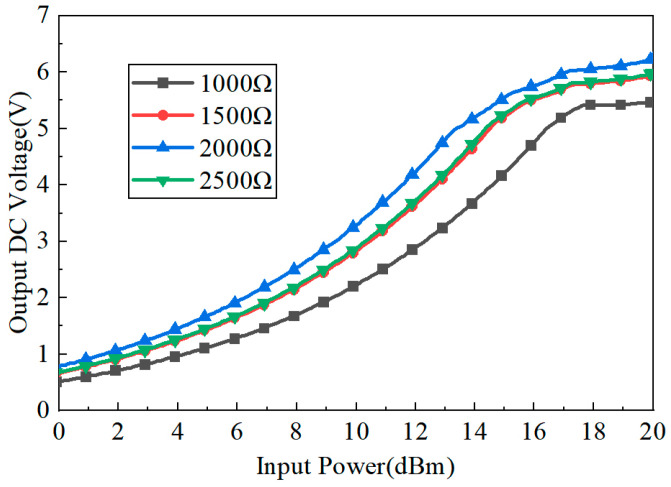
Output DC voltage versus input RF power *P_in_*.

As can be seen from Figure 4, the simulated output voltage is changed with the input power. For a different load *R*, the output DC voltage is different. As can be seen, the output DC voltage is increasing from 0.5 V to 6.3 V when the input power increases from 0 dB to 20 dB. When the input power *P_in_* is 13 dBm and the load *R* is 2000 Ω, the maximum output voltage is 4.92 V.

**Figure 5:**
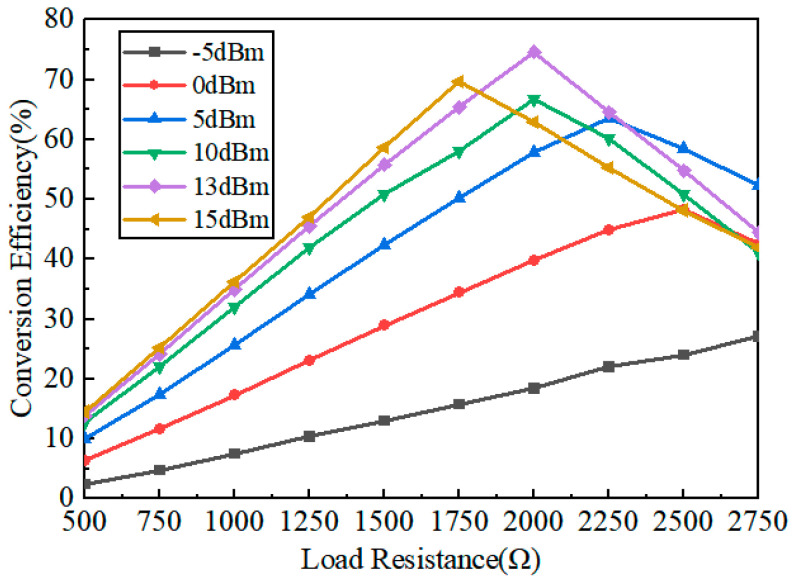
Conversion efficiency versus load resistance.

**Figure 6:**
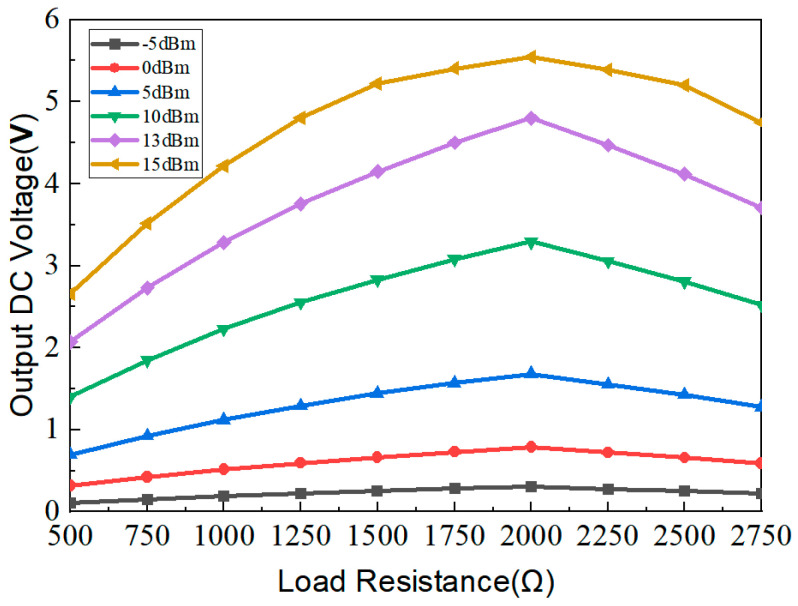
Output DC voltage versus load resistance.

In the previous rectification antenna system, the conversion efficiency is closely related to the load and the input power, and the conversion efficiency is not the same for different loads. As seen from Figure 5, for a low input power, such as −5 dBm, the conversion efficiency increases nearly linearly as the input power increases. For an input power larger than 0 dBm, the conversion efficiency increase first with the input power, and then the conversion efficiency decreases; each input power corresponds to a load value with a maximum conversion efficiency. The proposed rectifying circuit can maintain a high rectification efficiency of more than 60% over the load resistance range from 1500 Ω to 2000 Ω. Figure 6 shows the output voltage under different load resistances and input powers. As seen from Figure 6, for a low input power, such as −5 dBm, the output voltage of the circuit changes very slightly, even though the load increases. When the input power increases, the output voltage of the circuit increases with it. For an input power larger than 0 dBm, the output voltage increases first with the load, and then the output voltage decreases; each input power corresponds to a load value with a maximum output voltage. As can be seen, the maximum output voltage occurs at 2000 Ω when the input power is 15 dBm.

**Figure 7:**
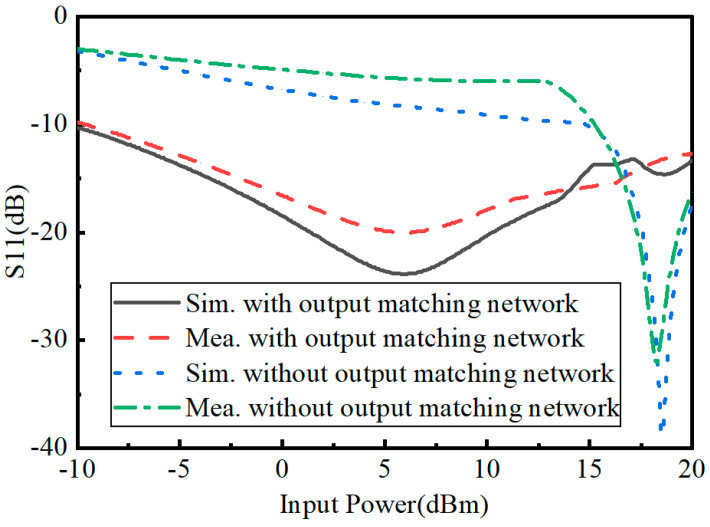
Measured and simulated S_11_ of the rectifiers with/without an output matching network versus input power.

As can be seen from Figure 7, by adding the output matching network, the input impedance can be adjusted. With the rectifying circuit with an output matching network, the measured results show an S_11_ less than −10 dB when the input power crosses from −9.6 dBm to −21.5 dBm. With the rectifying circuit without an output matching network, the measured S_11_ is less than −10 dB, using only the input power crossing from −14.8 dBm to −22.4 dBm. It is verified that the output matching network could expand the action operating range of the input power effectively.

## 3. Antenna Simulation and Design

**Figure 8:**
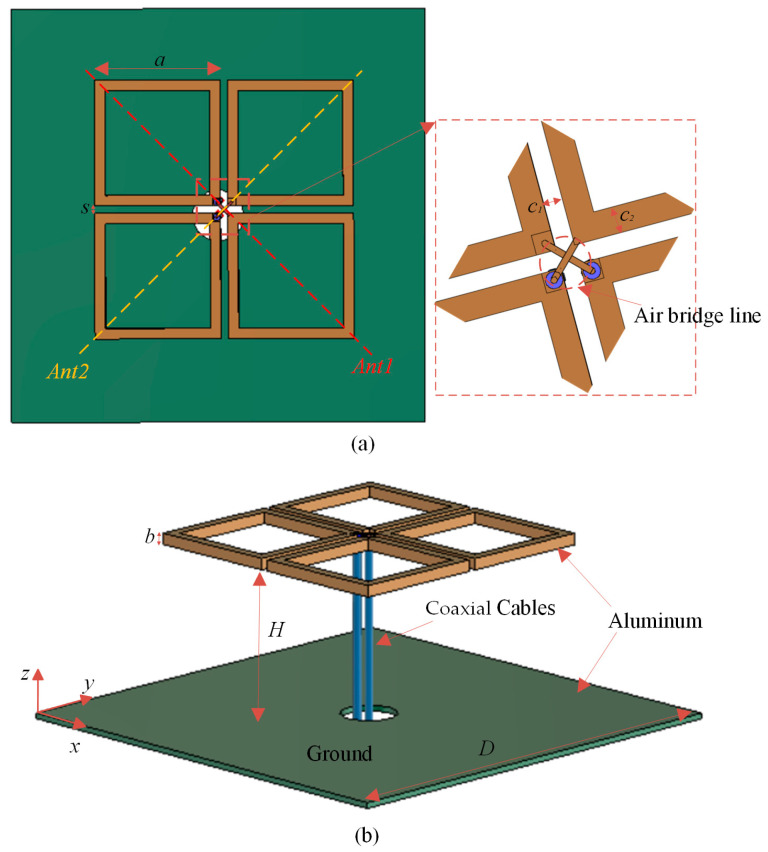
A 3D view of the proposed dual-polarized antenna. (**a**) Top view; (**b**) Side view.

To verify the proposed rectifying circuit, a broadband dual-polarized cross-dipole antenna with a vertical axial radiation pattern was designed. Figure 8 shows a 3D view of the proposed antenna. There are four identical square metal rings used as the radiators, where two square metal rings in the diagonal direction act as a dipole antenna. The double-linear polarization properties are obtained by orthogonally placing these two dipole antennas. The dipole antenna is fed by coaxial cable; the inner and external conductors of the coaxial line are connected to the two square metal rings (soldered via air bridge line), respectively. The coaxial feeder method guides the electromagnetic energy in the horizontal axial direction, and the maximum radiation pattern is pointed toward the vertical axial direction. Furthermore, for the dipole antenna located above the metal reflection ground, the vertical axial radiation is strengthened. The parameters of the proposed antenna are as follows: *H* = 32 mm, *D* = 80 mm, *a* = 24 mm, *b* = 2 mm, *s* = 1.4 mm, *c*_1_ = 2 mm and *c*_2_ = 2 mm.

**Figure 9:**
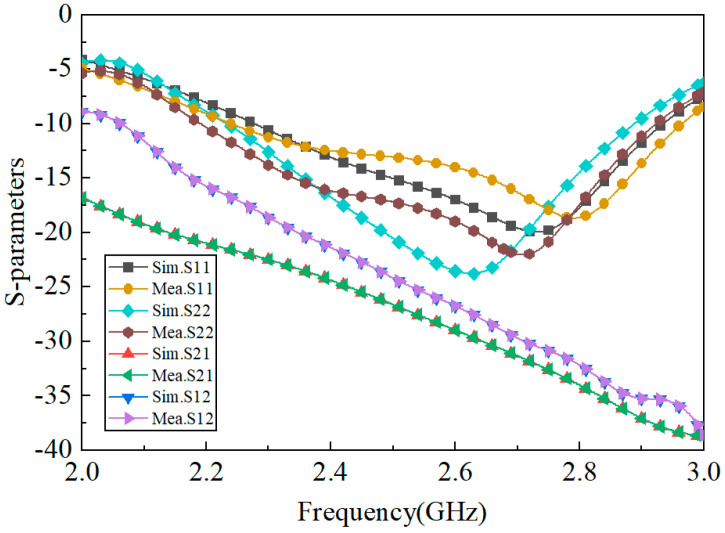
Simulated and measured S-parameters of the proposed antenna.

**Figure 10:**
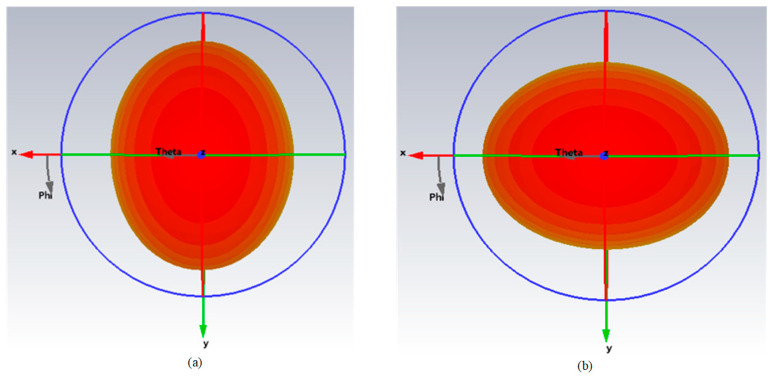
Simulated 3D radiation patterns of the proposed antenna. (**a**) Port 1 excited; (**b**) port 2 excited.

**Figure 11:**
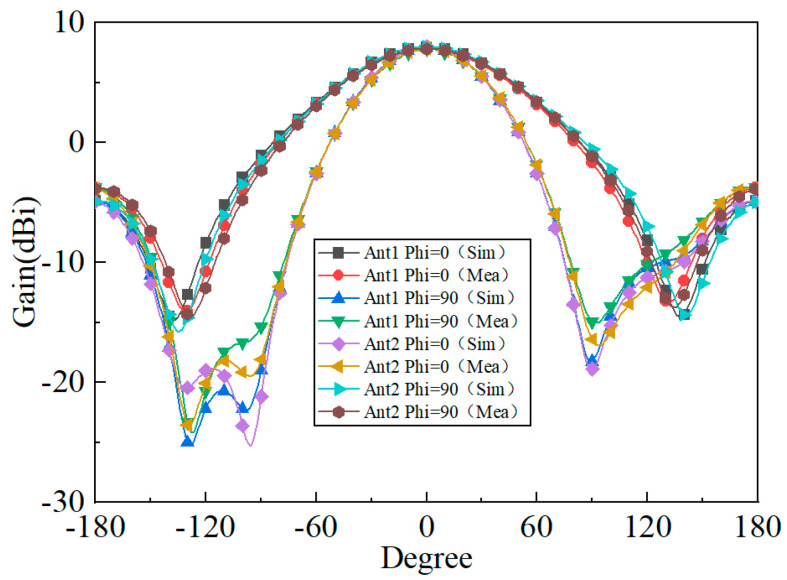
Simulated and measured 2D radiation patterns of the proposed antenna.

**Figure 12:**
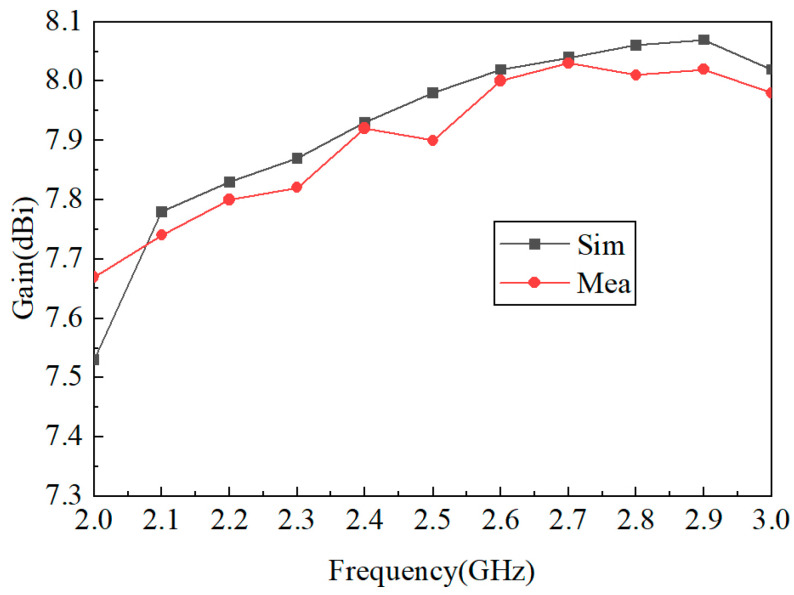
Simulated and measured gain of the proposed antenna.

Figure 9 shows the simulated and measured S-parameters of the proposed antenna; the relative impedance bandwidth (S_11_ ≤ −10 dB) covers from 2.3 to 2.9 GHz. Figure 10 shows the 3D simulated radiation patterns of the proposed antenna. As can be seen from Figure 10, the two polarized antenna patterns are mutually orthogonal; the proposed antenna has a stable directional pattern, which is suitable for point-to-point wireless power transmission. Figure 11 shows the simulated and measured 2D radiation patterns at 2.45 GHz, and the simulation and test results are relatively similar. Figure 12 shows the simulated and measured 2D radiation patterns of the proposed antenna; both of the polarized antennas have a gain of about 7.97 dBi.

## 4. Experiment Results

**Figure 13:**
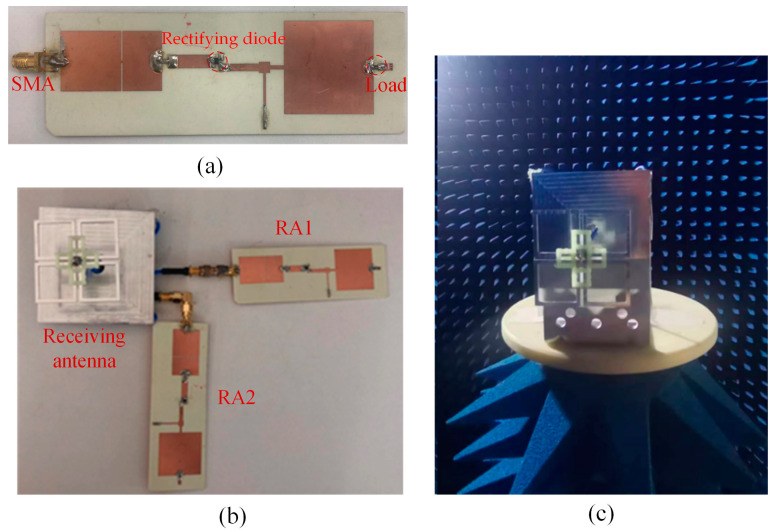
(**a**) Fabricated rectifying circuit. (**b**) Fabricated rectifying circuit connected with a dual-polarized antenna. (**c**) Antenna measurement setup.

Figure 13 shows the fabricated rectifying antenna system and the antenna test setup in an anechoic chamber. The antenna radiation patterns were measured using the far-field test method The rectifying circuit was fabricated on a PCB Rogers 4350B with a dielectric constant of 3.48 and loss tangent of 0.017. The whole size of the rectifying circuit is 110 mm × 34 mm × 1.524 mm; the circuit port is connected with an SMA connector.

The dual-polarized cross-dipole antenna was mainly made of aluminium. Two rigid coaxial cables were used to feed the antenna, each antenna port was wired to connect a rectifier circuit. Four plastic clips were used to fix the adjacent square metal rings to avoid shaking. The dual-polarized cross-dipole antenna was connected by two rectifying circuits through its two antenna ports; the two branches of the rectifying circuit are named RA1 and RA2, respectively.

**Figure 14:**
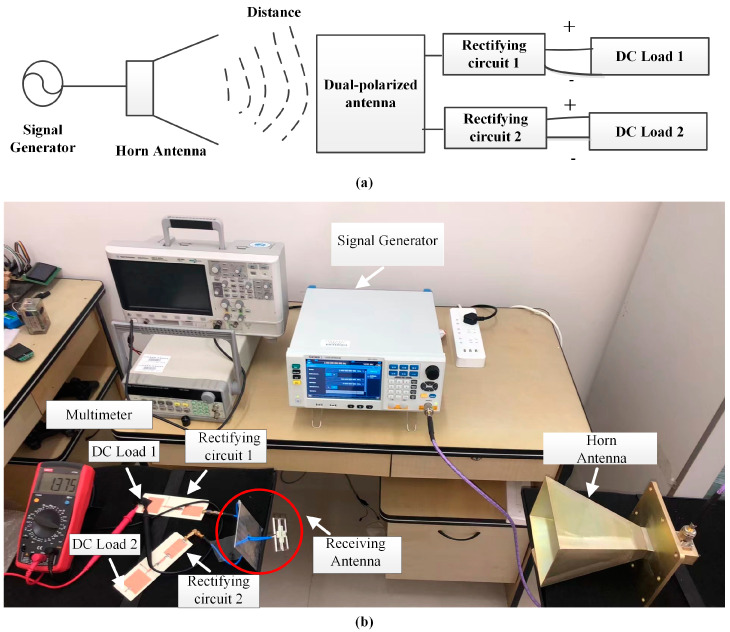
(**a**) Test schematic. (**b**) Measurement setup.

The wireless RF energy collection system experiment was conducted indoors; a measurement schematic and measurement setup are shown in Figure 14. The transmitting link consisted of a signal generator and transmitting horn antenna; the receiving link consisted of a dual-polarized cross-dipole antenna (receiving antenna), rectifying circuit and load.

Rectifying efficiency is one of the most important parameters for a rectifying circuit, which means the ability to convert the RF energy into DC power. The rectifying efficiency *η* can be calculated using the following equations:

(1) $$\eta =\frac{P_{out}}{P_{in}}=\frac{{V_{out}}^{2}/R_{L}}{P_{in}}\times 100\%$$

(2) $$P_{in}=P_{t}{\left(\frac{\lambda }{4\pi R}\right)}^{2}\mu G_{r}G_{t}$$

where *P_out_* is the DC output power, *P_in_* is the input RF power, *V_out_* is the output voltage, *R_L_* is the load of the rectifying circuit, *μ* is the polarization loss factor, *G_t_* and *G_r_* are the gains of the transmitting and receiving antennas, and *λ* is the free space wavelength of the operating frequency. In the actual test environment, the GIGOL DSG836A was used as the microwave source, the transmitting antenna used the standard gain horn, and the wireless RF energy collection system was placed in the far field of the horn antenna. The DC voltage output by the rectifying circuit in the wireless collecting system was measured on both ends of the resistive load (2000 Ω).

**Figure 15:**
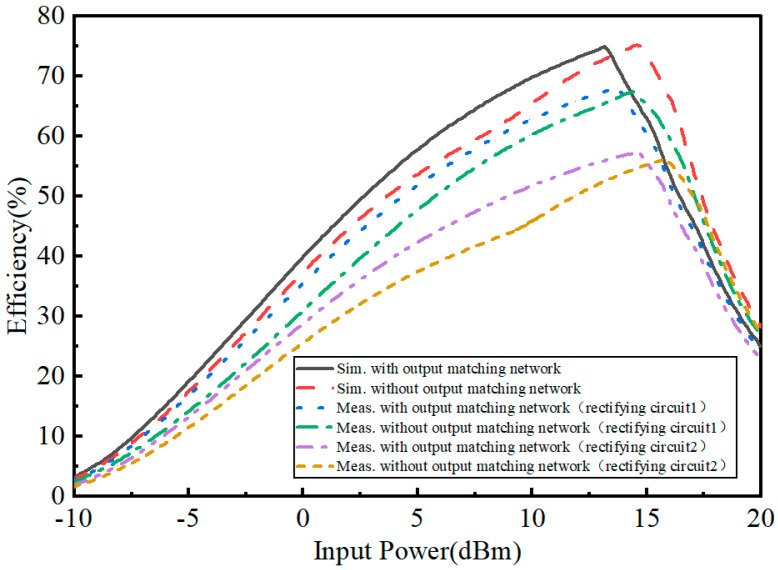
Simulated and measured efficiency of the rectifying circuit.

Figure 15 shows the simulated and measured efficiency of the rectifying circuit. In the simulation, the maximum efficiency reached 74.8% with the output matching network, when the input power was 13 dBm; the efficiency reached 74.9% without the output matching network, when the input power was 14.5 dBm. In the test, the efficiency of rectifier circuit 1 reached 68.03% with the output matching network, when the power was 13.6 dBm; the efficiency of rectifier circuit 1 reached 67.4% without the output matching network, when the power was 14.4 dBm. The efficiency of rectifier circuit 2 reached 57.2% with the output matching network, when the power was 14.5 dBm; the efficiency of rectifier circuit 2 reached 56% without the output matching network, when the power was 16 dBm.

In Table 2, the effect of the rectifier circuit designed in this study is compared with those of rectifier circuits published in the literature. Compared with reference [9], this work has the advantages of a higher rectifying efficiency and lower input power. Compared with reference [10], this work has the advantages of a lower input power and smaller size. Compared with reference [15], this work has the advantage of a lower input power. Compared with reference [19], this work has the advantage of a higher rectifying efficiency. In brief, it can be seen that the designed 2.45 GHz rectifier circuit shows a high conversion efficiency.

## 5. Conclusions

A 2.45 GHz high-efficiency rectifying circuit for a wireless RF energy collection system was proposed. The RF energy collection system is composed of a transmitting antenna, a receiving antenna, a rectifying circuit and a load. The designed receiving antenna is a kind of dual-polarized cross-dipole antenna; its bandwidth is 2.3–2.5 GHz and gain is 7.97 dBi. The proposed rectifying circuit adopts the technology of an output matching network, which can suppress the high-harmonic components. When the input power of 2.45 GHz is 13 dBm, and the load is 2000 Ω; the highest conversion efficiency of RF–DC is 74.8%, and the corresponding maximum DC output voltage is 4.92 V. The experiment results are in good agreement with the simulation results, which shows a good application prospect.

## Figures and Tables

**Table 1:** Optimized parameters of the proposed rectifying circuit.

Parameter	Unit	Parameter	Unit
L1	3 mm	W1	20 mm
L2	20 mm	W2	13 mm
L3	4.5 mm	W3	16 mm
L4	3.7 mm	W4	3.7 mm
L5	14.8 mm	W5	0.8 mm
L6	30.1 mm	W6	30.7 mm

**Table 2:** Rectifier performance comparison.

Ref.	Freq. (GHz)	S_11_ (dB)	Input Power (dBm)	Eff. (%)	Size of Rectenna (mm^2^)
[9]	2.45	−10	20	53.56	/
[10]	2.45	−24	20	/	160 × 130
[15]	2.45	−21	22	75.7	60 × 60
[19]	1.8/2.1/2.45	−24	0	63.1	80.4 × 128.9
This work	2.45	−10.5	13	74.8	120 × 40

## Data Availability

Data are contained within the article.
